# Seedy Toe

**Published:** 1893-02

**Authors:** Williamson Bryden


### SEEDY-TOE.

BY WILLIAMSON BRYDEN, V.S.

About fifteen months months ago I was called to examine a lame horse, one of a handsome pair of greys, used on the engine of the Fire Department at Medford, a pleasant town situated some five miles from Boston. They were a well-matched, nicely trained span of strong, speedy horses, some ten or eleven years old, fifteen hands three inches high, and weighing about fourteen hundred pounds each. Being general favorites, it was ill news for the boys when they learned that one of their splendid gray fire team was seriously lame.

On examination of the derelict foot—the off fore one—the disease was found to be what is generally known as "Seedy-toe." It had evidently been affected for many months, gradually showing greater brittleness of the lower part of the wall and of the sole in front. Indeed, the characteristic retrograde changes in the horn at the coronet in front, and its separation and projection nearer the toe, proclaimed the foot a victim of hoof depravity of many months existence; and the *stage* it had arrived at one which *Nature*, with all the assistance *Art* could give her, would require months to repair, unless a job of *patchwork* should be attempted and prove satisfactory to the owner.

The near foot was also imperfect, but from a different combination of circumstances. The wall had general contraction, but it had neither become *short and brittle* at the lower margin of each side of the hoof, nor was the growth at the coronet in front *almost entirely arrested* as shown by the other foot, two features of great interest and significance in the pathology of the horse's feet and limbs. Another point worthy of note suggested by the case is that (1) *Laminitis* followed by Seedy-toe may possibly be quite a different pathological condition, only one foot being usually affected; from (2) *Laminitis*, followed by chronic founder, in which two fore feet, two hind feet, or all four feet, are simultaneously and similarly affected. As both are curable conditions up to an advanced stage, and the treatment much alike, the importance of being able to discriminate between these two conditions is robbed of some of its practical value, but not of its scientific interest; that is whether one has an element of constitutional trouble which is wanting in the other.

In studies of this subject it is well to bear in mind that the hoofs of horses vary greatly in form, size and quality, in individuals even of the same family; and that domestication, with the restraints incident thereto, subjects them to many unfavorable experiences and *adverse* changes which still further *predispose* them to unsymmetrical growths, perhaps to diseases of the limbs, especially those peculiar to their species. For example, hoofs sometimes *do not grow alike*, *i. e.*, it is not unusual to find the two fore feet mismated, or the two hind ones either; indeed, all four feet may be different. Other circumstances, such as want of *wear and tear*, unsuitable soil and climate, accidents and exposures, especially during colthood, when the hoofs are growing, or rather developing, often inclines them to defective forms and qualities at maturity, which are readily excited to disease when the animal happens to be assigned to labor and surroundings unfavorable to his limbs. Seedy-toe is oftenest found affecting a fore foot, and predisposition to this family of diseases may be the result either of heredity or of some peculiarity acquired after birth.

There are many different *degrees* of such hoof *depravity*, as well as *stages* of *degeneration*, and it is in their repair rather than in their early history where their greatest resemblance is often seen.

1. One case may be nothing more than a small discolored area of sole where a nail has been driven too deep into a part where the vitality of the tissues has become so much impaired that they are unable to contribute their share either to their own nourishment or in repairing the injury.

2. Another case may be a foot with its hoof so warped and deformed that it resembles a *club foot;* a frequent *exciting* cause of this is when the coronet is crushed from a loaded team passing over it.

3. Another case may be one where the secreting structures, at the pyramidal process, are so crowded that the wall in front stops growing, and so does the laminæ extending to the toe. When these laminæ give way the toe of the coffin bone is forced down and back till it rests on the sole just in front of the frog; here it soon becomes bruised, then thin, and finally convex; the space between the coffin bone and the wall in front becomes filled with debris; the wall between the coronet and toe settles down, while the hoof at the heels grows vigorously, forcing the wings of the coffin bone upward and the pyramidal process forward against the defectively nourished wall in front, the toe of the coffin bone being held back by the perforatus tendom inserted in its sole. This variety has usually been described as an example of chronic founder *of one foot;* it is the form from which "Mortimer" suffered when Mr. Lorillard imported him from France. The following gives an outline of a case frequently met with, viz.:

When the sole has been dressed with a rasp the zone between the sole margin and the wall, which ought to be *white*, is found to be red. At next shoeing it will be a brown color; next it will be still browner, and so on till the wall and sole are entirely separated and crumbling. A gradual destructive change has been taking place within the foot where the circulation and other vital functions have been gradually becoming more disturbed and interfered with, perhaps for months. Accidents, such as burning with a hot shoe, must not be mistaken for this disease in its earliest stages.

As the coronet gradually tightens it exerts mechanical pressure on the coronary cushion and laminæ; the circulation is diminished in force and volume, and the wall shortens more and more from the extremities of its horn fibres, crumbling faster than it grows, till it cannot be reached by the nails. When the foot is without protection from either horn or shoe, it is unfit for work until nature has restored the part sufficiently to secure the shoe with nails.

The stage at which this disease has arrived indicates with considerable exactness both its age and the time required for its repair. As already stated, cases vary according as they reveal a history of gradual invasion, or evidence of having been hastened by some coincident, such as an accident, or from harsh or improper treatment.

With your permission I will now illustrate my remarks by describing the treatment of the Medford horse.

It was my good fortune to find the Superintendent, Mr. Arthur Symms, a gentleman of more than ordinary judgment in matters pertaining to the horse's feet and limbs. He did not expect me to perform a miracle, or to cure the beast in one or two times shoeing. After examining the case carefully, I assured him that complete recovery was possible, but that it would take at least eight months before he could again be used on the engine, as it would require that length of time before the hoof could grow down sufficiently for a shoe to be fastened so as to be equal to the quick, heavy work demanded in such a place. During most of the time, I further assured him, the horse could do enough slow, light work to pay for his keep, and as I would only require to see him about once a month, it would pay well to treat him rather than sacrifice a good animal by selling him for about $25, which was all anyone would be likely to give for him, with hardly three legs to stand on.

It was decided immediately to let me take charge of the case, so he was led into the shoeing shop, where I had the hoof trimmed as follows: From the coronet about half way to the toe the horn grew in narrow circles, gradually increasing towards the heels; below this, at the toe, the horn projected away from the coffin bone, the toe of which had crumbled away. The circles at the coronet in front, and the projecting horn lower down were all rasped and cut away till the part looked more like its natural shape.

This left the heels high, and the coffin bone in the position of an upright wedge, the diseased toe pointing to the sole in front of the frog, where the part was settled down. To correct this position of the coffin bone the commissures were pared out and the heels opened and lowered ; after this the wall was thinned at the heels and wherever else it could be done without interfering with the *nailing on of the shoe* as early as possible. Above the nail holes a *saw* was *used to cut the wall in line with the horn fibres ;* this instantly freed and limbered the crowding wall, and yet left enough hoof to nail to, which the rasp would have taken away. The saw is the same formerly used for "diamonding" the wall in cases of ringbone, side bones, enlarged cartileges, etc., cases where the hoof is always contracted. After carefully adjusting the hoof so as to set the coffin bone at an angle which would relieve the crowding of the pyramidal process and adjacent coffin bone against the horn in front, by allowing the wings of the coffin bone to settle between the hoof heels, a shoe was fitted as follows :

A plain, light shoe with a fairly wide web was taken, and from the toe to the second nail holes it was hammered out thin, and then rolled up in front of the foot to protect the part, which the disease had denuded of its horny protection, from coming accidentally in contact with the ground. Side lips, or clips, were turned up at each side to relieve some of the strain from the nails, and the nail holes were punched so as to be in line with and reach the wall, which was strongest. Two heel corks were then turned up about one-third of an inch high, and two more the same height, one on each side, set back from the toe about two inches ; when the shoe was applied he still walked lame on it, but after poulticing it for about three weeks, every night, with oil meal and 5 per cent. solution of carbolic acid, the hoof began to start growing, the sore spots to become less tender, and he was able to perform daily errands, and slow, light work.

As the Department buildings are near the marshy banks of Mystic River, he was allowed to stand in an open pen of fresh peat bog every day, for an hour or two, when not in poultices. The hoof was reduced every time he was shod, the wall grew down with perfect regularity, until at the conclusion of eight months, he was returned to his old place on the engine, as sound as ever. Twelve months after my treatment commenced, there was no trace of the disease, excepting a hollow place, or dent in the toe, showing where the point of the coffin bone had crumbled away from necrosis.

